# Mono and combination therapies in pulmonary arterial hypertension patients with comorbidities: A COMPERA analysis

**DOI:** 10.1002/ehf2.15254

**Published:** 2025-03-04

**Authors:** Dirk Skowasch, Christine Pausch, Doerte Huscher, David Pittrow, Judith Wede, Fabian Kreimendahl, Stephan Rosenkranz, Stephan Beckmann, Matthias Held, Ekkehard Grünig, H. Ardeschir Ghofrani, Hans Klose, Andris Skride, Michael Halank, Stefan Stadler, Marion Delcroix, Anton Vonk‐Noordegraaf, Ralf Ewert, Grzegorz Kopec, Marius M. Hoeper, Karen M. Olsson

**Affiliations:** ^1^ Department of Internal Medicine II Bonn Germany; ^2^ GWT‐TUD GmbH, Innovation Center Real World Evidence Dresden Germany; ^3^ Institute of Biometry and Clinical Epidemiology Berlin Institute of Health, Charité‐Universitätsmedizin Berlin Germany; ^4^ Institute for Clinical Pharmacology, Medical Faculty Technical University Dresden Germany; ^5^ Janssen‐Cilag GmbH Neuss Germany; ^6^ Clinic III for Internal Medicine (Cardiology) and Center for Molecular Medicine (CMMC), and the Cologne Cardiovascular Research Center (CCRC) University of Cologne Cologne Germany; ^7^ Specialist Clinic Löwenstein Löwenstein Germany; ^8^ Department of Internal Medicine, Respiratory Medicine and Ventilatory Support Medical Mission Hospital, Central Clinic Würzburg Germany; ^9^ Translational Lung Research Center Heidelberg (TLRC), German Center for Lung Research (DZL) Center for Pulmonary Hypertension, Thoraxklinik at Heidelberg University Hospital Heidelberg Germany; ^10^ Department of Internal Medicine, Justus‐Liebig‐University Giessen Universities of Giessen and Marburg Lung Center (UGMLC) Giessen Germany; ^11^ German Center of Lung Research (DZL) Giessen Germany; ^12^ Department of Medicine Imperial College London London UK; ^13^ Department of Respiratory Medicine Eppendorf University Hospital Hamburg Germany; ^14^ Riga Stradiņš University, Riga, Latvia; Rare Diseases Unit VSIA Pauls Stradins Clinical University Hospital Riga Latvia; ^15^ Medical Clinic and Polyclinic I University Hospital Carl Gustav Carus of the Technical University of Dresden Dresden Germany; ^16^ Department of Internal Medicine II University Medical Center Regensburg Regensburg Germany; ^17^ Clinical Department of Respiratory Diseases University Hospitals of Leuven and Laboratory of Respiratory Diseases and Thoracic Surgery (BREATHE), Department of Chronic Diseases and Metabolism (CHROMETA), KU Leuven ‐ University of Leuven Leuven Belgium; ^18^ Department of Pulmonary Medicine, Amsterdam Cardiovascular Sciences Amsterdam UMC, Vrije Universiteit Amsterdam Amsterdam Netherlands; ^19^ Department of Respiratory Medicine, Clinic of Internal Medicine University Medical Center Greifswald Greifswald Germany; ^20^ Department of Cardiac and Vascular Diseases Jagiellonian University Medical College, John Paul II Hospital in Krakow Krakow Poland; ^21^ Department of Respiratory Medicine and Infectious Diseases and German Center of Lung Research (DZL) Hannover Medical School Hannover Germany

**Keywords:** COMPERA database, initial monotherapy and combination therapy, matched‐pair analysis, PAH and comorbidities

## Abstract

**Aims:**

Pulmonary arterial hypertension (PAH) is often diagnosed in elderly patients with comorbidities. Although initial monotherapy is recommended for these patients, the value of combination therapy remains unclear. Here, we compare the efficacy of initial monotherapy and combination therapy in PAH patients with cardiovascular comorbidities.

**Methods and results:**

Data from adult patients with incident pre‐capillary PAH and cardiovascular comorbidities from the COMPERA database (European registry for PH) were analysed. A matched‐pair analysis of patients treated with monotherapy versus combination therapy based on age, sex, WHO functional class (FC) and 4‐strata risk at baseline was performed. The matching strategy identified 216 pairs of PAH patients with cardiovascular comorbidities, who differed considerably from the enrolled patient population (*n* = 1871), especially in terms of mean age (mono: matched pairs 62.9 ± 13.5 years vs. 70.6 ± 11.4 years, combination: matched pairs 62.0 ± 13.6 years vs. 60.5 ± 14.9 years). In the matched‐pair analysis, the initial combination therapy group showed more pronounced improvements in WHO‐FC, N‐terminal pro–B‐type natriuretic peptide (BNP/NT‐proBNP) and risk status than patients treated with initial monotherapy, with no significant differences in 6‐min walk distance (6MWD), PAH‐related hospitalisations, survival and drug discontinuation.

**Conclusions:**

This analysis suggests that PAH patients with comorbidities may benefit more pronounced from combination therapy regarding WHO‐FC, BNP/NT‐pro‐BNP and risk status without a significant difference in survival. Good tolerability is indicated. However, given the relatively younger patient matched subgroup, these findings may not necessarily apply to older patients with a wider range of comorbidities.

## Graphical abstract

**AIM** This analysis provides a comparison of the efficacy of initial mono and combination therapy in patients with PAH and cardiovascular comorbidities or with risk factors of HFpEF (heart failure with preserved ejection fractioin).**METHOD** Data from the European COMPERA registry were used for a matched pair analysis. Changes in clinical parameters, risk status and PAH‐related hospitalizations, survival and continuation of PAH treatment from baseline to follow‐up were assessed.**FINDINGS** 216 matched pairs of patients were identified. This cohort differed from the original one, especially in terms of age. At follow‐up patients with initial combination therapy showed greater improvements e.g. in risk status (fig.). No significant differences in survival were observed.**Figure:** Risk stratification at first follow‐up among patients with at least one cardiovascular comorbidity.

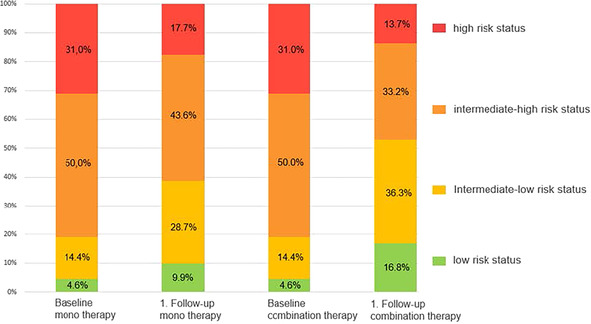

## Introduction

Pulmonary arterial hypertension (PAH) affects the small pulmonary arteries through vasoconstriction and vascular cell proliferation, leading to remodelling and an increased pulmonary vascular resistance. The increased right ventricular afterload results in right ventricular failure and may ultimately lead to death.^1^, ^2^, ^3^ PAH can be divided into different subtypes according to different aetiologies including idiopathic PAH (IPAH),^3^ which is used to categorise patients with pre‐capillary pulmonary hypertension of unknown origin. IPAH was originally observed mainly in young, otherwise healthy individuals, particularly females.^3^, ^4^ Meanwhile, several phenotypes of patients with IPAH are emerging. Recent data from the United States and Europe suggest that PAH is now frequently diagnosed in elderly patients (i.e., those aged ≥ 65 years) who often present with cardiopulmonary comorbidities and risk factors for left heart disease.^3^ In a cluster analysis of the European COMPERA (Comparative, Prospective Registry of Newly Initiated Therapies for Pulmonary Hypertension) registry, the median age of patients newly diagnosed with PAH was 72 years and more than 80% had comorbidities.^5^, ^6^, ^7^ The management of elderly patients with PAH and comorbidities remains unclear. The 2022 ESC/ERS guidelines provide an algorithm for the treatment of patients with PAH.^3^ This algorithm distinguishes between PAH with and without cardiopulmonary comorbidities. In contrast to the recommendations for upfront combination therapies in patients with PAH without comorbidities, a less aggressive approach is recommended for PAH patients with cardiopulmonary comorbidities, owing to a lack of supportive evidence for the use of initial combination therapy in this group of patients. Registry data suggest that elderly PAH patients with comorbidities are less likely to receive combination therapy than younger ones without comorbidities.^4^, ^5^, ^8^, ^9^ A recent COMPERA analysis found that most patients with comorbidities were treated with phosphodiesterase 5‐inhibitors (PDE5i) monotherapy, while only about 33% of the patients received combination therapy within 1 year after diagnosis (vs. 62% of patients without comorbidities).^10^ So far, there is a growing number of studies focusing on these PAH patients with comorbidities, respectively examining data through post hoc analyses, notably because such patients may respond less well to targeted therapies and may display reduced drug tolerability.^9^, ^11^, ^12^, ^13^ To gain further insight into this aspect, a comparison of PAH patients with comorbidities receiving either monotherapy or combination therapy would be helpful. To account for inherent differences in these two patient populations, a matched pair analysis was used to compare the effectiveness of initial monotherapy and combination therapy in patients with PAH and cardiovascular comorbidities.

## Methods

### Database

Details of COMPERA (www.COMPERA.org; registered at Clinicaltrials.gov under the identifier NCT01347216) and in the European Medicines Agency Real World Data Catalogue under 1000000103) have been previously reported.^4^, ^5^, ^14^ The ongoing and completely web‐based pulmonary hypertension (PH) registry was established in 2007. Today, expert sites from 12 European countries participate in the registry, with most of the patients included in the registry (approx. 80%) coming from German centres. COMPERA is one of the largest PH registries and currently (June 2024) has enrolled about 12 900 patients, including more than 7500 patients with PAH. Baseline, follow‐up and outcome data from patients who receive targeted therapies for PH are collected in COMPERA. The registry is researcher‐initiated and funded by the pharmaceutical industry. COMPERA has been approved by the ethics committees of all participating centres and assures data protection. Written informed consent from patients is obtained prior to inclusion.

### Patients

**Figure 1:**
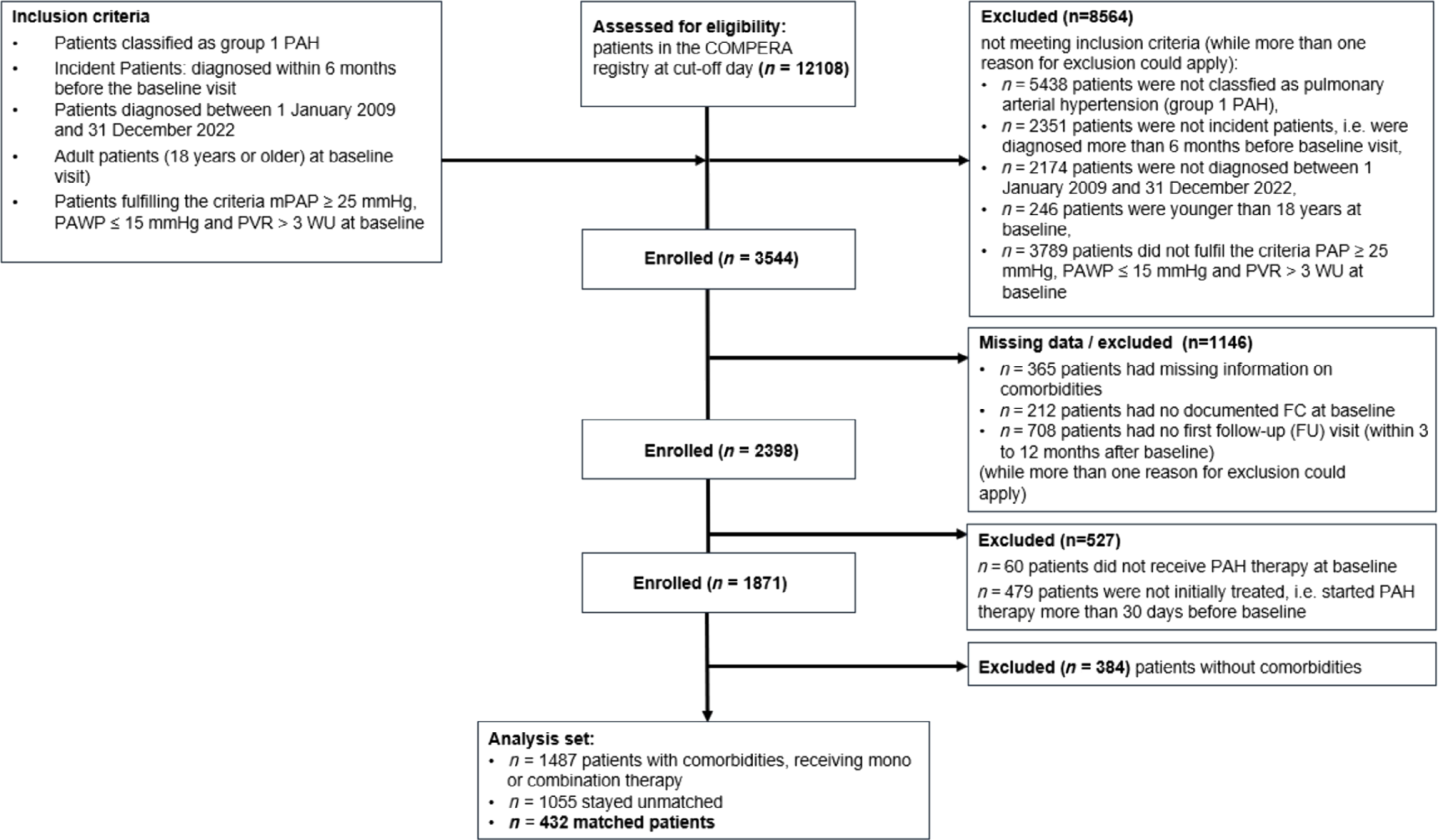
Strobe diagram of patient cohort selection process. FC, World Health Organization (WHO) Functional Class (FC); mmHg, millimetres of mercury; mPAP, mean pulmonary arterial pressure; PAH, pulmonary arterial hypertension; PAWP, pulmonary artery wedge pressure; PVR, pulmonary vascular resistance.

For the present analysis, patients were selected by the following criteria: Diagnosis of PAH between 1 January 2009 and 31 December 2022 and within 6 months before baseline visit; mean pulmonary arterial pressure (mPAP) ≥ 25 mmHg, pulmonary arterial wedge pressure (PAWP) ≤ 15 mmHg and pulmonary vascular resistance (PVR) > 3 Wood Units (WU) at baseline; adult patients (≥18 years at baseline visit); WHO FC available at baseline; information on cardiovascular comorbidities documented; at least one documented follow‐up visit within 3 to 12 months after treatment start (*Figure* *1*).

### Definitions

#### Baseline visit

The baseline visit represents data from treatment‐naïve patients with newly diagnosed PAH (within 6 months) at the time of PAH therapy initiation.

#### First follow‐up visit

The first follow‐up visit was defined as earliest follow‐up visit within 3 to 12 months after initiation of PAH therapy (i.e., baseline).

#### Comorbidities

Comorbidity status was recorded at baseline visit. The following cardiovascular comorbidities were considered: obesity (body mass index (BMI) ≥ 30 kg/m^2^), coronary heart disease, arterial hypertension and diabetes mellitus. Patients with at least one of these comorbidities were defined as ‘with comorbidities’.

#### Mono/combination therapy

Two cohorts of patients with cardiovascular comorbidities (as described above) were defined: as either receiving monotherapy or combination therapy within 30 days after baseline. Combination therapy refers to any combination of the following PH agents: endothelin receptor antagonists (ERA), PDE‐5i, a soluble guanylate cyclase stimulator (sGC‐S), prostacyclin analogues or prostacyclin receptor agonists (PCA). Calcium channel blockers (CCB) were not considered. Patients who were classified as vasoresponders and treated with CCB only were excluded from this analysis.

### Matching and statistical analyses

We performed a matched‐pair analysis (following general matching principles^15^) in which patients were matched in a 1:1 manner, without replacement, using the R package MatchIt (Nonparametric Preprocessing for Parametric Causal Inference).^16^ Each patient from the monotherapy group was matched to a partner from the combination therapy group based on the following four categorical criteria:

- age at baseline (18–44 years, 45–64 years, 65–74 years and ≥75 years)
- sex (male and female)
- WHO FC at baseline (I/II and III/IV)
- 4‐strata risk status at baseline (low, intermediate‐low, intermediate‐high, and high)

Matching was performed using an exact matching approach with greedy matching, meaning that once a pair of patients was matched, they were no longer available for the matching procedure. The division of patients by the matching criteria allowed for a perfect 1:1 matching of patients, because all four matching variables were categorical.

Effectiveness outcomes were changes in WHO FC, 6MWD, NT‐proBNP and risk status from baseline at first follow‐up. Long‐term outcome measurements included time to death, time to first PAH‐related hospitalisation and time to discontinuation of PAH medications. No missing data were inputed.

Continuous numeric variables were summarised using mean, standard deviation (SD) and median [Q1, Q3]. Categorical variables were presented as frequency counts including absolute and relative frequencies. Change in parameters was calculated for patients with at least two assessments by building the difference of values between time points.

Cox proportional hazards models and the Kaplan–Meier method were used to analyse time‐to‐event data. In cases the event of interest did not occur, patients were censored at the day of last contact. When analysing transplant‐free survival, transplanted patients were censored at the day of transplantation. The statistical software package R version 4.1.3.1. was used for all statistical analyses.

## Results

### Patient characteristics

Out of 1871 newly diagnosed PAH patients, 384 had no comorbidities at baseline and were excluded. Of the remaining 1487 patients with PAH and cardiovascular comorbidities (*Figure* *1*, ‘pre‐matched’ in the following), 432 patients were selected for analysis based on the matching variables described: 216 patients receiving monotherapy were matched to 216 patients receiving combination therapy. The characteristics of these patients are shown in *Table* *1*.

**Table 1:** Patient characteristics, hemodynamic parameters and comorbidities at baseline, and FC, 6MWD, BNP/NT‐proBNP, risk status at baseline and first follow‐up/therapy switch from baseline to first follow up and drug class at first follow up of all matched patients

	Monotherapy *n* = 216		Combination therapy *n* = 216	
	Mean (SD), median or *n* (%)	Missing, *n* (%)	Mean (SD), median or *n* (%)	Missing, *n* (%)
Age (years) at baseline	62.9 (13.5), 64.0	0 (0.0%)	62.0 (13.6), 63.0	0 (0.0%)
18 to 44 years	25 (11.6%)		25 (11.6%)	
45 to 64 years	89 (41.2%)		89 (41.2%)	
65 to 74 years	62 (28.7%)		62 (28.7%)	
75 years or over	40 (18.5%)		40 (18.5%)	
Sex		0 (0.0%)		0 (0.0%)
Female	146 (67.6%)		146 (67.6%)	
Male	70 (32.4%)		70 (32.4%)	
Number of comorbidities at baseline, category		38 (17.6%)		20 (9.3%)
0	0 (0.0%)		0 (0.0%)	
1–2	130 (73.0%)		160 (81.6%)	
3–4	48 (27.0%)		36 (18.4%)	
Obesity at baseline	115 (53.2%)	0 (0.0%)	110 (50.9%)	0 (0.0%)
Coronary heart disease at baseline	83 (44.9%)	31 (14.4%)	49 (24.3%)	14 (6.5%)
Arterial hypertension at baseline	135 (70.7%)	25 (11.6%)	151 (72.2%)	7 (3.2%)
Diabetes mellitus at baseline	69 (35.9%)	24 (11.1%)	59 (28.5%)	9 (4.2%)
RAP (mmHg) at baseline	9.5 (5.8), 9.0	19 (8.8%)	9.6 (5.3), 9.0	28 (13.0%)
PAP (mmHg) at baseline	47.7 (11.7), 47.0	0 (0.0%)	49.0 (10.9), 48.0	0 (0.0%)
PCWP (mmHg) at baseline	9.9 (3.4), 10.0	0 (0.0%)	9.2 (3.4), 9.0	0 (0.0%)
PVR (WU) at baseline	9.6 (4.7), 8.9	0 (0.0%)	10.7 (4.6), 10.0	0 (0.0%)
CI (L/min/m^2^) at baseline	2.3 (0.7), 2.2	31 (14.4%)	2.1 (0.7), 2.0	15 (6.9%)
SvO_2_ (%) at baseline	63.2 (9.0), 63.0	48 (22.2%)	60.1 (9.7), 61.0	39 (18.1%)
DLCO (% pred.) ) at baseline	53.3 (41.1), 46.5	98 (45.4%)	49.5 (21.4), 45.0	51 (23.6%)
FC at baseline		0 (0.0%)		0 (0.0%)
I	0 (0.0%)		0 (0.0%)	
II	29 (13.4%)		29 (13.4%)	
III	156 (72.2%)		156 (72.2%)	
IV	31 (14.4%)		31 (14.4%)	
FC at first follow‐up		43 (19.9%)		35 (16.2%)
I	2 (1.2%)		7 (3.9%)	
II	39 (22.5%)		58 (32.0%)	
III	115 (66.5%)		104 (57.5%)	
IV	17 (9.8%)		12 (6.6%)	
6MWD (m) at baseline	279.9 (130.3), 283.0	63 (29.2%)	272.6 (136.7), 275.0	57 (26.4%)
6MWD (m) at first follow‐up	321.1 (118.4), 330.0	70 (32.4%)	327.5 (128.7), 335.0	71 (32.9%)
BNP (ng/L) at baseline	442 (532), 269	145 (67.1%)	442 (380), 333	185 (85.6%)
BNP (ng/L) at first follow‐up	425 (1372), 143	163 (75.5%)	241 (240), 148	193 (89.4%)
NT‐proBNP (ng/L) at baseline	3492 (6914), 1850	90 (41.7%)	4708 (11981), 2007	47 (21.8%)
NT‐proBNP (ng/L) at first follow‐up	2084 (4251), 879	98 (45.4%)	1889 (4677), 526	57 (26.4%)
Risk at baseline		0 (0.0%)		0 (0.0%)
Low	10 (4.6%)		10 (4.6%)	
Intermediate‐low	31 (14.4%)		31 (14.4%)	
Intermediate‐high	108 (50.0%)		108 (50.0%)	
High	67 (31.0%)		67 (31.0%)	
Risk at first follow‐up		35 (16.2%)		26 (12.0%)
Low	18 (9.9%)		32 (16.8%)	
Intermediate‐low	52 (28.7%)		69 (36.3%)	
Intermediate‐high	79 (43.6%)		63 (33.2%)	
High	32 (17.7%)		26 (13.7%)	
Switch from baseline to first follow‐up		0 (0.0%)		0 (0.0%)
Monotherapy to combination therapy	53 (24.5%)		0 (0.0%)	
Combination to monotherapy	0 (0.0%)		15 (6.9%)	
No switch	154 (71.3%)		198 (91.7%)	
Switch to no therapy	9 (4.2%)		3 (1.4%)	
Drug class at first follow‐up^*^				
ERA	99 (45.8%)	0 (0.0%)	189 (87.5%)	0 (0.0%)
PDE5i	148 (68.5%)	0 (0.0%)	188 (87.0%)	0 (0.0%)
PCA	6 (2.8%)	0 (0.0%)	49 (22.7%)	0 (0.0%)
sGC	12 (5.6%)	0 (0.0%)	20 (9.3%)	0 (0.0%)

6MWD, 6 min walk distance; BNP, B‐type natriuretic peptide; DLCO (% pred.) , diffusing capacity factor of the lung for carbon monoxide (predictive value; ERA, endothelium receptor antagonist; FC, functional class; NT‐proBNP, N‐terminal pro–B‐type natriuretic peptide; PDE5i, phosphodiesterase‐5 inhibitor; PAP, pulmonary artery pressure; PAWP, pulmonary arterial wedge pressure; PCA, prostacyclin analogues or prostacyclin receptor agonists; RAP, right atrial pressure; PVR (WU) , pulmonary vascular resistance (wood units); sGC, soluble guanylate cyclase stimulator; SvO_2_, oxygen saturation.

^a^ Multiple answers possible.

One thousand thirty patients receiving monotherapy and 25 patients receiving combination therapy remained without a matching partner. Before matching, the average age in the monotherapy group was 70.6 years, whereas patients who received a combination therapy were about 10 years younger (average 60.5 years). After the matching process, the mean age was 62.9 and 62.0 years, respectively. In addition, there were differences in the incidence of cardiovascular diseases and obesity between the matched patients (especially in the monotherapy group) and the pre‐matched patients (*Table* *1*). The full characteristics of all patients with cardiovascular comorbidities, as well as of the patients with and without matching partners at baseline are summarised in *Table* *S1*.

The majority of matched patients in both groups did not have a change in therapy (mono, combination or no therapy) from baseline to first follow‐up within 3 to 12 months (median time from start of treatment to first FU was 4.5 months in the monotherapy group and 4.8 months in the combination therapy‐group): 24.5% of patients with monotherapy switched to combination therapy (pre‐matched: 18.5%) and 6.9% of the patients with combination therapy switched back to monotherapy (pre‐matched: 7.5%). No switch was recorded for 71.3% of the monotherapy group (pre‐matched: 78.1%) and for 91.7% in the combination therapy group (pre‐matched: 91.3%).

The main drug classes received by matched patients with combination therapy at first follow‐up were ERA (87.5%) and PDE5i (87.0%). Patients on monotherapy received mainly PDE5i (68.5%).

### FC, 6MWD, NT‐proBNP and risk status at baseline and first follow‐up

**Figure 2:**
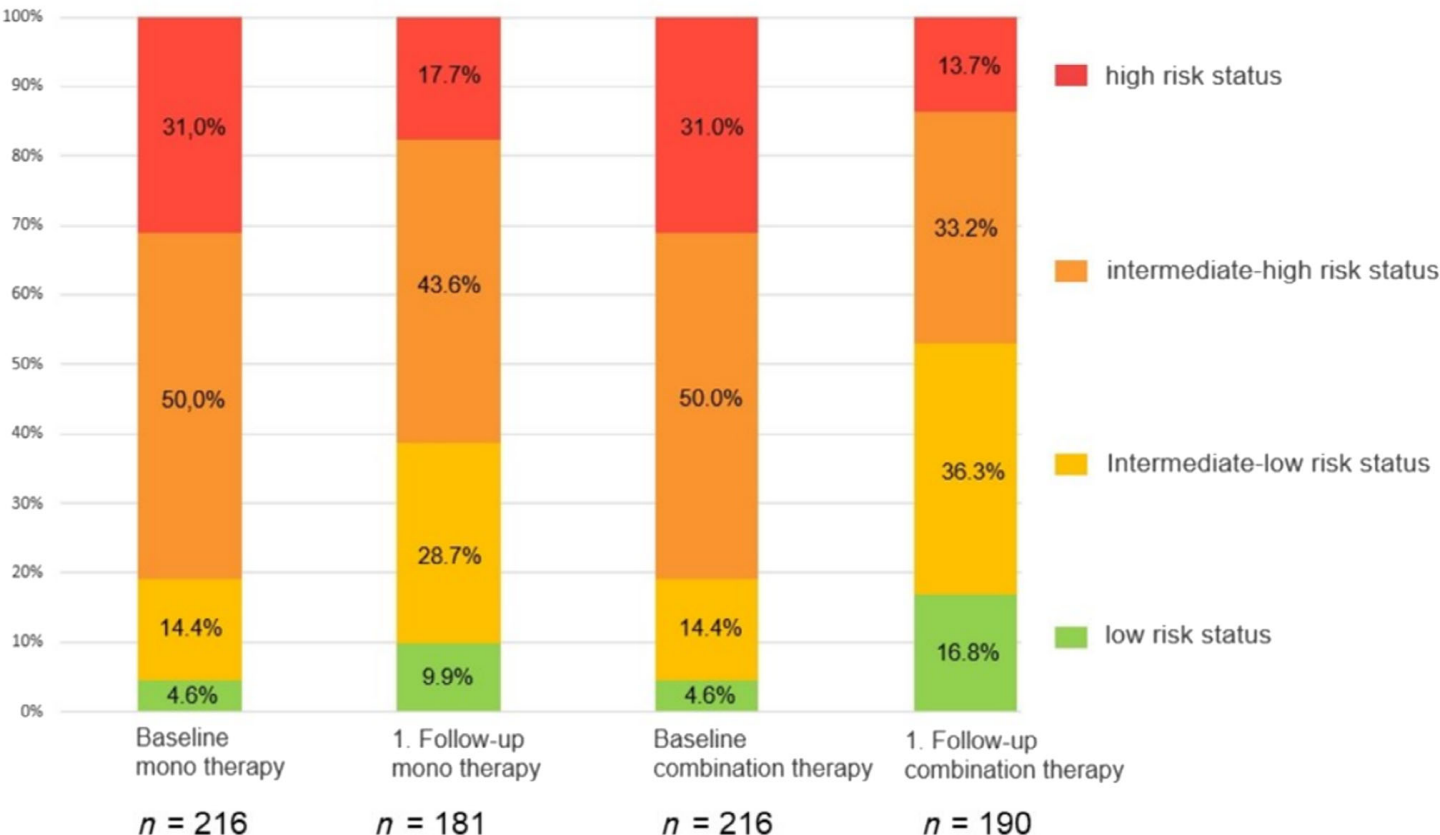
Risk stratification at first follow‐up according to initial treatment strategy among patients with at least one cardiovascular comorbidity (after matching on age, sex, FC and risk status).

Changes from baseline to first follow‐up are shown in *Table* *2*. There were significantly larger improvements with combination therapy compared to monotherapy in FC, NT‐proBNP and risk status (*Figure* *2*), but not in 6MWD. The full data set of all patients with cardiovascular comorbidities, as well as patients with and without matching partners is provided in the supplement (*Table* *S2*).

**Table 2:** Outcomes of all matched patients with cardiovascular comorbidities, monotherapy versus combination therapy. No. %, mean (SD) or median [Q1, Q3]

	Monotherapy	Combination therapy	*P*‐value
Change in FC from baseline to first FU^f^			0.0299^a^
Improvement	43 (24.9%)	67 (37.0%)	
Stable	111 (64.2%)	102 (56.4%)	
Worsening	19 (11.0%)	12 (6.6%)	
Change in 6MWD from baseline to first FU^b^	37.7 (96.8)	53.3 (98.0)	0.2246^c^
Relative change in NT‐proBNP/BNP from baseline to first FU^d^	−28.3 [−72.3, 66.9]	−57.0 [−83.9, −6.8]	<0.0001^e^
Change in risk status from baseline to first FU^g^			0.0285^a^
Improvement	71 (39.2%)	100 (52.6%)	
Stable	93 (51.4%)	73 (38.4%)	
Worsening	17 (9.4%)	17 (8.9%)	

6MWD, 6 min walk distance; BNP, B‐type natriuretic peptide; FC, functional class; FU, follow up; NT‐proBNP, N‐terminal pro–B‐type natriuretic peptide.

^a^ Chi‐squared test.

^b^ Positive values indicate a longer distance at first follow‐up.

^c^ Welch *t*‐test.

^d^ Values in percent, positive value indicate higher values at first follow‐up.

^e^ Wilcoxon test.

^f^ From 173 patients in the monotherapy group and 181 in the combination therapy group, post‐baseline values for FC were available and calculation done for change in FC.

^g^ From 181 patients in the monotherapy group and 190 in the combination therapy group, post‐baseline values for risk status were available and calculation done for change in risk status.

### Survival and PAH‐related hospitalisations

**Figure 3:**
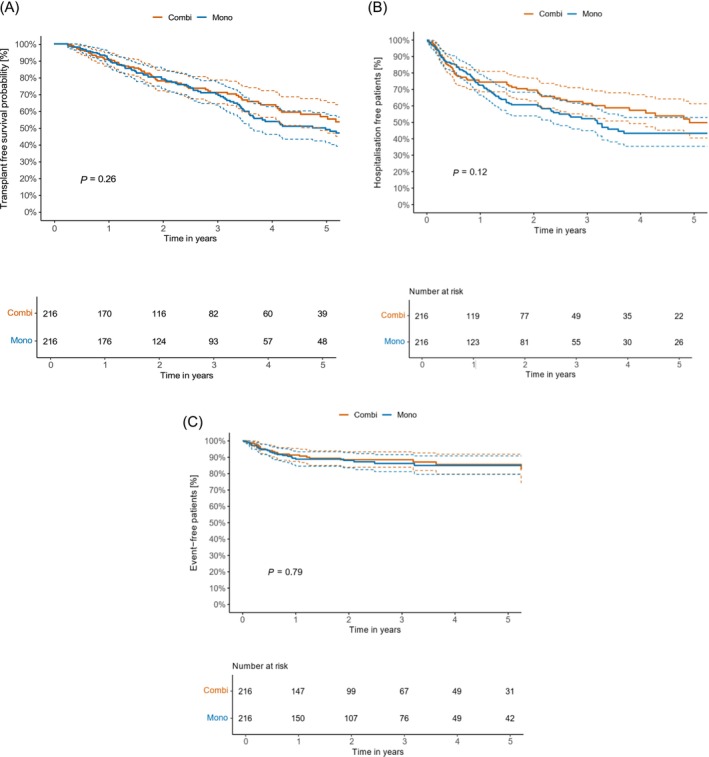
(A) Kaplan–Meier curves with 95% confidence intervals for transplant‐free survival time of all matched patients with comorbidities, monotherapy versus combination therapy. Survival estimates at 1 year are 91.3% (95% CI [87.5; 95.2]%) for monotherapy group and 90.7% (95% CI [86.8; 94.8]%) for combination therapy group. Survival estimates at 3 years are 70.2% (95% CI [63.6; 77.4]%) for the monotherapy group and 71.1% (95% CI [64.4; 78.5]%) for the combination therapy group. The survival estimates at 5 years are 49.0% (95% CI [41.1; 58.3]%) for monotherapy group and 56.7% (95% CI [48.5; 66.3]%) for the combination therapy group. (B) Kaplan–Meier curves with 95% confidence intervals for time to PH related hospitalisation of all matched patients with comorbidities, monotherapy versus combination therapy. Survival estimates at 1 year are 72.6% (95% CI [66.6; 79.1]%) for monotherapy group and 74.5% (95% CI [68.6; 80.9]%) for combination therapy group. Survival estimates at 3 years are 52.3% (95% CI [45.0; 60.8]%) for monotherapy group and 61.4% (95% CI [53.9; 70.1]%) for combination therapy group. Survival estimates at 5 years are 43.3% (95% CI [35.5; 52.9]%) for monotherapy group and 49.8% (95% CI [40.5; 61.3]%) for combination therapy group. (C) Kaplan–Meier curves with 95% confidence intervals for time to first discontinuation of therapy (monotherapy to no therapy and combination to monotherapy) of all matched patients with comorbidities, monotherapy versus combination therapy.

The transplant‐free survival was comparable in both cohorts (*Figure* *3A*, *P* = 0.26). The Kaplan–Meier estimates for transplant‐free survival at 1, 3 and 5 years for patients with monotherapy were 91.3%, 70.2% and 49.0%, and 90.7%, 71.1% and 56.7% with combination therapy. In addition, there was no significant difference in PAH‐related hospitalisations (*Figure* *3B*, *P* = 0.12). Hospitalisation‐free rates at 1, 2 and 3 years for patients with monotherapy were 72.6%, 52.3% and 43.3% and 74.5%, 61.4% and 49.8% with combination therapy.

### Treatment continuations

Continuation rates of PAH treatments are depicted in *Figure* *3C* (*P* = 0.79). Continuation rates at 1, 2 and 3 years for patients with comorbidities and monotherapy were 88.8%, 88.0% and 86.2%, respectively. In patients with combination therapy, the corresponding numbers were 91%, 86% and 86%.

## Discussion

In this matched‐pair analysis of patients with newly diagnosed PAH receiving monotherapy or combination therapy as initial treatment, we found more pronounced improvements in WHO FC, BNP/NT‐proBNP and risk status in patients treated with combination therapy at first follow up, while there were no significant differences in 6MWD, while no difference could be seen in up to 5 years survival, hospitalisation and continuation rates of PAH treatment between the two groups based on initial therapy strategy only (not taking into account possible therapy escalation).

So far, evidence supporting the safety, tolerability and efficacy of initial combination therapy in patients with PAH and comorbidities remains limited. In a post‐hoc analysis of the AMBITION study,^11^ the primary analysis set (patients without multiple risk factors for left heart disease, *n* = 500) was compared with the ex‐primary analysis set (patients with multiple risk factors for left heart disease, *n* = 105). In both sets, patients received either initial combination therapy using ambrisentan and tadalafil or monotherapy with each of these compounds. The time from randomisation to first clinical failure event was defined as primary endpoint. In both sets, patients with combination therapy showed numerically greater treatment effect compared to monotherapy. However, the effect was attenuated and not statistically significant in the ex‐primary set. This is in line with further post‐hoc analyses and registries^9^, ^12^, ^13^ showing that PAH patients with comorbidities may respond less well to targeted therapies and may display a reduced drug tolerability; while randomised controlled trials are missing, patient's characteristics in this present analysis of the COMPERA registry closely resemble those observed in randomised controlled trials.

To our knowledge, the present report is the first that describes long term survival comparing the effectiveness of initial monotherapy and combination therapy in patients with PAH and cardiovascular comorbidities. Of note, there was no difference in survival probability between both groups, despite more pronounced improvements in WHO FC, BNP/NT‐proBNP and risk status in patients treated with combination therapy at first follow up. In contrast, another COMPERA analysis showed that improvements in FC were associated with improved consecutive survival, while improvements in NT‐proBNP had relatively little and improvements in 6MWD no predictive value.^17^ In addition, improvements in multicomponent endpoints and risk stratification tools were associated with better long‐term survival—without considering patients with comorbidities separately.^17^ In the meantime, it has been shown that the 4‐strata risk tool predicted outcome also in patients with IPAH irrespective of the presence of comorbidities.^10^

Even if PH was the leading cause of death also in PAH patients with comorbidities,^10^ one may speculate that IPAH patients with comorbidities are often likely to die from the comorbidities and not from right heart failure alone. In addition, the current analysis showed similar treatment discontinuation rates, although previous studies and registries suggested increased side effects and poorer drug tolerability.

There are limitations to our analysis common for registry settings such as post hoc nature lacking prospective confirmation, the possibility of misclassification bias or undocumented comorbidities and short follow‐up period but most importantly the selection bias resulting from the matching process. Real‐world experience shows that mainly young patients with cardiovascular comorbidities receive combination therapy, which is confirmed by our data: the mean age in the monotherapy group of pre‐matched patients was 70.6 years, whereas patients who received combination therapy were about 10 years younger, with an mean age of 60.5 years. In addition, there are significant differences (*Table* *1*) in the incidence of cardiovascular disease, diabetes and obesity between the matched patients (especially in the monotherapy group) and the pre‐matched patients, which may also lead to statistical biases. Hence, the remaining small group of matched pairs cannot be considered representative for the adult PAH patient population with cardiovascular comorbidities we initially selected.

However, it should be noted that this analysis was not designed to be representative of both groups and thus to be able to draw an absolute conclusion on the treatment benefit. Rather, the question of whether or not patients with comparable characteristics benefit more from the combination therapy is of interest in everyday clinical practice.

In conclusion, our data suggest that selected patients with PAH and cardiovascular comorbidities may derive some benefit from initial PAH combination therapy, as greater improvements were observed in WHO FC, BNP/NT‐pro‐BNP levels and risk status compared to monotherapy. However, there were no significant differences between the two therapy regimens in 6MWD, PAH‐related hospitalisations and transplant‐free survival thereby confirming also the recent recommendations of 7th world conference of PH^18^ that has not included a separate ‘comorbidities’ arm in the PAH treatment algorithm. Further data including RCTs are needed to determine the clinical value of initial combination therapy in patients with PAH and comorbidities.

## Conflict of interest

Dirk Skowasch received fees for lectures and/or consulting and/or research support to institution from AstraZeneca, Boehringer Ingelheim, Chiesi, GSK, Janssen Pharmaceutical Companies of Johnson & Johnson, MSD, Sanofi, Pfizer, BMBF and DFG. Doerte Huscher none declared. Christine Pausch none declared. David Pittrow received fees for consultations from Actelion, Amgen, Aspen, Bayer, Biogen, Boehringer Ingelheim, Daiichi Sankyo, MSD, Novartis, Sanofi‐Genzyme, Takeda, Viatris and Zambon. Judith Wede is currently employed as Scientific Manager Therapeutic Area PAH at Janssen‐Cilag GmbH, a Johnson & Johnson company. Fabian Kreimendahl is currently employed as Scientific Specialist Medical Evidence Generation at Janssen‐Cilag GmbH, a Johnson & Johnson company. Stephan Rosenkranz has received fees for lectures and/or consultations from Abbott, Acceleron, Actelion, Bayer, Bristol‐Myers Squibb, Gilead, GlaxoSmithKline, Janssen, MSD, Novartis, Pfizer, United Therapeutics and Vifor; research grants to the institution from AstraZeneca, Actelion, Bayer Janssen and Novartis. Stephan Beckmann has received speaker fees from AstraZeneca and travel support from Janssen Cilag. Matthias Held has received speaker fees and honoraria for consultations from Actelion, Bayer, Boehringer Ingelheim Pharma, GlaxoSmithKline, Janssen, MSD, Novartis, Pfizer, Nycomed, Roche and Servier. Ekkehard Grünig served as speaker and/or consultant honoraria for Bayer Healthcare, Ferrer, GEBRO, GlaxoSmithKline, Janssen Biotech, Merck Sharp & Dohme (MSD) and OMT outside the submitted work. H. Ardeschir Ghofrani has received honoraria for consultations and/or speaking at conferences from Bayer HealthCare AG, Actelion, Encysive, Pfizer, Ergonex, Eli Lilly and Novartis; is a member of advisory boards for Acceleron, Bayer HealthCare AG, Pfizer, GlaxoSmithKline, Actelion, Eli Lilly, Merck, Encysive and Ergonex and has received governmental grants from the German Research Foundation, Excellence Cluster Cardiopulmonary Research, State Government of Hessen and the German Ministry for Education and Research. Hans Klose has received speaker fees and honoraria for consultations from Actelion, Bayer, GlaxoSmithKline, Janssen, MSD, Novartis, Pfizer and United Therapeutics. Andris Skride served as speaker and/or consultant for KRKA, Gossamer Bio, AOP Orphan and Novartis. Michael Halank received fees for lectures and/or consulting from AOP, AstraZeneca, Janssen Pharmaceutical Companies of Johnson & Johnson and MSD. Stefan Stadler received fees for lectures and/or consulting and/or research support to institution from AOP Health, Bayer, Gossamer Bio, Janssen‐Cilag GmbH, Keros Therapeutics, MSD and Pfizer. Marion Delcroix has received an institutional research grant from Janssen; institutional speaker and consultant fees from Bayer, MSD, Acceleron, AOP, Ferrer, Daiichi Sankyo, Inari, Janssen and Pfizer outside the submitted work and is holder of the Janssen Chair for Pulmonary Hypertension at KU Leuven. Anton Vonk‐Noordegraaf is supported by the Netherlands CardioVascular Research Initiative (CVON‐2012–08 PHAEDRA, CVON‐2017–10 DOLPHIN‐GENESIS) and the Netherlands Organization for Scientific Research (NWO‐VICI: 918.16.610). In addition his institute received speakers money from Johnson & Johnson, MSD, Actelion, Bayer and Ferrer in the past 3 years. Finally he served as a member of the scientific advisory board of Morphogen‐X, Ferrer, Gosammer Bio Services Inc, Altavant, MSD and Johnson & Johnson. Ralf Ewert received fees for lectures and/or consulting from LungPacer, OMT, AOP Orphan, AstraZeneca, Boehringer Ingelheim, Janssen Pharmaceutical and Berlin Chemie. Grzegorz Kopec served as consultant or speaker for Acceleron Pharma, Janssen, AOP Orphan, GossamerBio, Pfizer and Merck (paid to self). Marius M. Hoeper served as consultant or speaker for Acceleron Pharma, Inc., Actelion Pharmaceuticals, AOP Orphan, Bayer Healthcare, Ferrer, GossamerBio, Janssen Global Services, LLC and Merck & Co., Inc. (Rahway, NJ, USA) (paid to self). Karen M. Olsson served as consultant or speaker for Acceleron Pharma, Inc., Actelion Pharmaceuticals, AOP Orphan, Bayer Healthcare, Ferrer, GossamerBio, Janssen Global Services, LLC and Merck & Co., Inc. (Rahway, NJ, USA) (paid to self).

## Supporting information


**Table S1.** patient characteristics at baseline.
**Table S2.** FC, 6MWD, NT‐proBNP, risk status and therapy switch/drug class from baseline to first follow‐up.
