# Supplementary material for: Mono and combination therapies in pulmonary arterial hypertension patients with comorbidities: A COMPERA analysis
Source: ESC Heart Fail. 2025 Mar 4;12(4):2726–35. doi: 10.1002/ehf2.15254 (PMC12287794; doi:10.1002/ehf2.15254)
Supplement: Supplementary file 1 — Table S1. patient characteristics at baseline. Table S2. FC, 6MWD, NT‐proBNP, risk status and therapy switch/drug class from baseline to first follow‐up. [file EHF2-12-2726-s001.docx]

***Table S1 patient characteristics at baseline***

| Table S1: patient characteristics at baseline: matched patients / patients without matching partners / all patients with comorbidities |
| --- |

| characteristics | matched patients with  comorbidities (n=216)  Mono therapy  Mean (SD), Median  or No. (%) | Missing (%) | matched patients  with comorbidities (n=216)  Combination therapy  Mean (SD), Median  or No. (%) | Missing (%) | patients without matching  partner with comorbidities  (n=1030)  Mono therapy  Mean (SD), Median  or No. (%) | Missing (%) | patients without  matching partner  with comorbidities  (n=25)  Combination  therapy  Mean (SD),  Median  or No. (%) | Missing (%) | all patients with comorbidities  (n=1246)  Mono therapy  Mean (SD), Median  or No. (%) | Missing (%) | all patients with comorbidities  (n=241)  Combination therapy  Mean (SD), Median  or No. (%) | Missing (%) |
| --- | --- | --- | --- | --- | --- | --- | --- | --- | --- | --- | --- | --- |
| Age (years)  18 to 44 years  45 to 64 years  65 to 74 years  75 years or over  Sex  Female  Male  BMI (kg/m^2^)  Country  Germany  Non-German | **62.9 (13.5), 64.0**  **25 (11.6%)**  **89 (41.2%)**  **62 (28.7%)**  **40 (18.5%)**  **146 (67.6%)**  **70 (32.4%)**  **31.1 (6.9), 30.8**  **166 (76.9%)**  **50 (23.1%)** | **0 (0.0%)**  **0 (0.0%)**  **0 (0.0%)**  **0 (0.0%)** | **62.0 (13.6), 63.0**  **25 (11.6%)**  **89 (41.2%)**  **62 (28.7%)**  **40 (18.5%)**  **146 (67.6%)**  **70 (32.4%)**  **31.0 (6.4), 30.1**  **179 (82.9%)**  **37 (17.1%)** | **0 (0.0%)**  **0 (0.0%)**  **0 (0.0%)**  **0 (0.0%)** | **72.2 (10.2), 75.0**  **15 (1.5%)**  **185 (18.0%)**  **307 (29.8%)**  **523 (50.8%)**  **628 (61.0%)**  **402 (39.0%)**  **29.6 (6.3), 28.8**  **974 (94.6%)**  **56 (5.4%)** | **0 (0.0%)**  **0 (0.0%)**  **0 (0.0%)**  **0 (0.0%)** | **48.0 (19.6), 49.0**  **12 (48.0%)**  **8 (32.0%)**  **3 (12.0%)**  **2 (8.0%)**  **18 (72.0%)**  **7 (28.0%)**  **32.5 (8.4), 33.5**  **22 (88.0%)**  **3 (12.0%)** | **0 (0.0%)**  **0 (0.0%)**  **0 (0.0%)**  **0 (0.0%)** | **70.6 (11.4), 74.0**  **40 (3.2%)**  **274 (22.0%)**  **369 (29.6%)**  **563 (45.2%)**  **774 (62.1%)**  **472 (37.9%)**  **29.8 (6.4), 28.9**  **1140 (91.5%)**  **106 (8.5%)** | **0 (0.0%)**  **0 (0.0%)**  **0 (0.0%)**  **0 (0.0%)** | **60.5 (14.9), 63.0**  **37 (15.4%)**  **97 (40.2%)**  **65 (27.0%)**  **42 (17.4%)**  **164 (68.0%)**  **77 (32.0%)**  **31.1 (6.7), 30.3**  **201 (83.4%)**  **40 (16.6%)** | **0 (0.0%)**  **0 (0.0%)**  **0 (0.0%)**  **0 (0.0%)** |
| PAH classification  1.1 Idiopathic  1.2 Heritable  1.3 Drug-associated  1.4.1 Associated with CTD  1.4.2 Associated with HIV infection  1.4.3 Associated w. portal hyperten.  1.4.4 Associated with CHD  1.5 PVOD/PCH | **138 (63.9%)**  **7 (3.2%)**  **3 (1.4%)**  **38 (17.6%)**  **2 (0.9%)**  **16 (7.4%)**  **8 (3.7%)**  **4 (1.9%)** | **0 (0.0%)** | **129 (59.7%)**  **5 (2.3%)**  **5 (2.3%)**  **53 (24.5%)**  **1 (0.5%)**  **13 (6.0%)**  **1 (0.5%)**  **9 (4.2%)** | **0 (0.0%)** | **780 (75.7%)**  **10 (1.0%)**  **9 (0.9%)**  **144 (14.0%)**  **4 (0.4%)**  **48 (4.7%)**  **27 (2.6%)**  **8 (0.8%)** | **0 (0.0%)** | **18 (72.0%)**  **1 (4.0%)**  **1 (4.0%)**  **2 (8.0%)**  **1 (4.0%)**  **0 (0.0%)**  **2 (8.0%)**  **0 (0.0%)** | **0 (0.0%)** | **918 (73.7%)**  **17 (1.4%)**  **12 (1.0%)**  **182 (14.6%)**  **6 (0.5%)**  **64 (5.1%)**  **35 (2.8%)**  **12 (1.0%)** | **0 (0.0%)** | **147 (61.0%)**  **6 (2.5%)**  **6 (2.5%)**  **55 (22.8%)**  **2 (0.8%)**  **13 (5.4%)**  **3 (1.2%)**  **9 (3.7%)** | **0 (0.0%)** |
| FC  I  II  III  IV  6MWD (m)  BNP (ng/L)  NT-proBNP (ng/L) | **0 (0.0%)**  **29 (13.4%)**  **156 (72.2%)**  **31 (14.4%)**  **279.9 (130.3), 283.0**  **442 (532), 269**  **3492 (6914), 1850** | **0 (0.0%)**  **63 (29.2%)**  **145 (67.1%)**  **90 (41.7%)** | **0 (0.0%)**  **29 (13.4%)**  **156 (72.2%)**  **31 (14.4%)**  **272.6 (136.7), 275.0**  **442 (380), 333**  **4708 (11981), 2007** | **0 (0.0%)**  **57 (26.4%)**  **185 (85.6%)**  **47 (21.8%)** | **3 (0.3%)**  **103 (10.0%)**  **777 (75.4%)**  **147 (14.3%)**  **276.9 (105.2), 282.0**  **301 (354), 174**  **2834 (4997), 1405** | **0 (0.0%)**  **225 (21.8%)**  **892 (86.6%)**  **273 (26.5%)** | **0 (0.0%)**  **6 (24.0%)**  **9 (36.0%)**  **10 (40.0%)**  **244.0 (222.1), 300.0**  **NA (NA), NA**  **4208 (6779), 2371** | **0 (0.0%)**  **18 (72.0%)**  **25 (100.0%)**  **12 (48.0%)** | **3 (0.2%)**  **132 (10.6%)**  **933 (74.9%)**  **178 (14.3%)**  **277.4 (109.6), 282.0**  **349 (427), 187**  **2928 (5314), 1491** | **0 (0.0%)**  **288 (23.1%)**  **1037 (83.2%)**  **363 (29.1%)** | **0 (0.0%)**  **35 (14.5%)**  **165 (68.5%)**  **41 (17.0%)**  **271.4 (140.4), 277.5**  **442 (380), 333**  **4673 (11675), 2038** | **0 (0.0%)**  **75 (31.1%)**  **210 (87.1%)**  **59 (24.5%)** |
| Risk  Low  Intermediate-low  Intermediate-high  High | **10 (4.6%)**  **31 (14.4%)**  **108 (50.0%)**  **67 (31.0%)** | **0 (0.0%)** | **10 (4.6%)**  **31 (14.4%)**  **108 (50.0%)**  **67 (31.0%)** | **0 (0.0%)** | **17 (1.8%)**  **209 (21.6%)**  **521 (53.9%)**  **219 (22.7%)** | **64 (6.2%)** | **1 (7.7%)**  **3 (23.1%)**  **2 (15.4%)**  **7 (53.8%)** | **12 (48.0%)** | **27 (2.3%)**  **240 (20.3%)**  **629 (53.2%)**  **286 (24.2%)** | **64 (5.1%)** | **11 (4.8%)**  **34 (14.8%)**  **110 (48.0%)**  **74 (32.3%)** | **12 (5.0%)** |
| Comorbidities  No  Yes  Number of comorbidities  0  1  2  3  4  Number of comorbidities, categ.  0  1-2  3-4  Obesity  Coronary heart disease  Arterial hypertension  Diabetes mellitus  Hemodynamic parameters:  RAP (mmHg)  PAP (mmHg)  PAWP (mmHg)  PVR (WU)  CI (L/min/m2)  SvO2 (%)  DLCO (% pred.)  Therapy  No therapy  Mono therapy  Combination therapy  Drug class ∗  ERA  PDE5i  PCA  sGC | **0 (0.0%)**  **216 (100.0%)**    **0 (0.0%)**  **64 (36.0%)**  **66 (37.1%)**  **37 (20.8%)**  **11 (6.2%)**    **0 (0.0%)**  **130 (73.0%)**  **48 (27.0%)**  **115 (53.2%)**  **83 (44.9%)**  **135 (70.7%)**  **69 (35.9%)**  **9.5 (5.8), 9.0**  **47.7 (11.7), 47.0**  **9.9 (3.4), 10.0**  **9.6 (4.7), 8.9**  **2.3 (0.7), 2.2**  **63.2 (9.0), 63.0**  **53.3 (41.1), 46.5**    **0 (0.0%)**  **216 (100.0%)**  **0 (0.0%)**  **67 (31.0%)**  **135 (62.5%)**  **0 (0.0%)**  **14 (6.5%)** | **0 (0.0%)**  **38 (17.6%)**  **38 (17.6%)**  **0 (0.0%)**  **31 (14.4%)**  **25 (11.6%)**  **24 (11.1%)**  **19 (8.8%)**  **0 (0.0%)**  **0 (0.0%)**  **0 (0.0%)**  **31 (14.4%)**  **48 (22.2%)**  **98 (45.4%)**  **0 (0.0%)**  **0 (0.0%)**  **0 (0.0%)**  **0 (0.0%)**  **0 (0.0%)** | **0 (0.0%)**  **216 (100.0%)**  **0 (0.0%)**  **99 (50.5%)**  **61 (31.1%)**  **26 (13.3%)**  **10 (5.1%)**  **0 (0.0%)**  **160 (81.6%)**  **36 (18.4%)**  **110 (50.9%)**  **49 (24.3%)**  **151 (72.2%)**  **59 (28.5%)**  **9.6 (5.3), 9.0**  **49.0 (10.9), 48.0**  **9.2 (3.4), 9.0**  **10.7 (4.6), 10.0**  **2.1 (0.7), 2.0**  **60.1 (9.7), 61.0**  **49.5 (21.4), 45.0**  **0 (0.0%)**  **0 (0.0%)**  **216 (100.0%)**  **202 (93.5%)**  **195 (90.3%)**  **30 (13.9%)**  **17 (7.9%)** | **0 (0.0%)**  **20 (9.3%)**  **20 (9.3%)**  **0 (0.0%)**  **14 (6.5%)**  **7 (3.2%)**  **9 (4.2%)**  **28 (13.0%)**  **0 (0.0%)**  **0 (0.0%)**  **0 (0.0%)**  **15 (6.9%)**  **39 (18.1%)**  **51 (23.6%)**  **0 (0.0%)**  **0 (0.0%)**  **0 (0.0%)**  **0 (0.0%)**  **0 (0.0%)** | **0 (0.0%)**  **1030 (100.0%)**  **0 (0.0%)**  **356 (37.7%)**  **323 (34.2%)**  **205 (21.7%)**  **60 (6.4%)**  **0 (0.0%)**  **679 (71.9%)**  **265 (28.1%)**  **438 (42.5%)**  **333 (34.3%)**  **849 (84.2%)**  **373 (37.6%)**  **7.9 (4.5), 7.0**  **40.1 (10.5), 38.0**  **9.6 (3.4), 10.0**  **8.2 (4.2), 7.2**  **2.2 (0.7), 2.1**  **63.3 (8.0), 64.0**  **50.5 (22.5), 47.0**  **0 (0.0%)**  **1030 (100.0%)**  **0 (0.0%)**  **137 (13.3%)**  **869 (84.4%)**  **0 (0.0%)**  **24 (2.3%)** | **0 (0.0%)**  **86 (8.3%)**  **86 (8.3%)**  **0 (0.0%)**  **59 (5.7%)**  **22 (2.1%)**  **38 (3.7%)**  **75 (7.3%)**  **0 (0.0%)**  **0 (0.0%)**  **0 (0.0%)**  **38 (3.7%)**  **119 (11.6%)**  **257 (25.0%)**  **0 (0.0%)**  **0 (0.0%)**  **0 (0.0%)**  **0 (0.0%)**  **0 (0.0%)** | **0 (0.0%)**  **25 (100.0%)**  **0 (0.0%)**  **11 (47.8%)**  **8 (34.8%)**  **4 (17.4%)**  **0 (0.0%)**  **0 (0.0%)**  **19 (82.6%)**  **4 (17.4%)**  **16 (64.0%)**  **4 (17.4%)**  **16 (69.6%)**  **5 (21.7%)**  **11.6 (5.7), 12.0**  **56.4 (18.1), 54.0**  **9.2 (4.0), 9.0**  **13.1 (5.9), 14.8**  **2.2 (1.1), 1.9**  **57.4 (10.8), 56.0**  **52.9 (21.1), 50.0**  **0 (0.0%)**  **0 (0.0%)**  **25 (100.0%)**  **24 (96.0%)**  **25 (100.0%)**  **2 (8.0%)**  **0 (0.0%)** | **0 (0.0%)**  **2 (8.0%)**  **2 (8.0%)**  **0 (0.0%)**  **2 (8.0%)**  **2 (8.0%)**  **2 (8.0%)**  **3 (12.0%)**  **0 (0.0%)**  **0 (0.0%)**  **0 (0.0%)**  **0 (0.0%)**  **5 (20.0%)**  **9 (36.0%)**  **0 (0.0%)**  **0 (0.0%)**  **0 (0.0%)**  **0 (0.0%)**  **0 (0.0%)** | **0 (0.0%)**  **1246 (100.0%)**  **0 (0.0%)**  **420 (37.4%)**  **389 (34.7%)**  **242 (21.6%)**  **71 (6.3%)**  **0 (0.0%)**  **809 (72.1%)**  **313 (27.9%)**  **553 (44.4%)**  **416 (36.0%)**  **984 (82.1%)**  **442 (37.3%)**  **8.1 (4.8), 8.0**  **41.4 (11.1), 40.0**  **9.7 (3.4), 10.0**  **8.4 (4.3), 7.4**  **2.2 (0.7), 2.1**  **63.3 (8.2), 64.0**  **50.9 (25.7), 47.0**  **0 (0.0%)**  **1246 (100.0%)**  **0 (0.0%)**  **204 (16.4%)**  **1004 (80.6%)**  **0 (0.0%)**  **38 (3.0%)** | **0 (0.0%)**  **124 (10.0%)**  **124 (10.0%)**  **0 (0.0%)**  **90 (7.2%)**  **47 (3.8%)**  **62 (5.0%)**  **94 (7.5%)**  **0 (0.0%)**  **0 (0.0%)**  **0 (0.0%)**  **69 (5.5%)**  **167 (13.4%)**  **355 (28.5%)**  **0 (0.0%)**  **0 (0.0%)**  **0 (0.0%)**  **0 (0.0%)**  **0 (0.0%)** | **0 (0.0%)**  **241 (100.0%)**  **0 (0.0%)**  **110 (50.2%)**  **69 (31.5%)**  **30 (13.7%)**  **10 (4.6%)**  **0 (0.0%)**  **179 (81.7%)**  **40 (18.3%)**  **126 (52.3%)**  **53 (23.6%)**  **167 (72.0%)**  **64 (27.8%)**  **9.8 (5.4), 9.0**  **49.8 (12.0), 48.0**  **9.2 (3.5), 9.0**  **11.0 (4.8), 10.3**  **2.1 (0.7), 2.0**  **59.8 (9.8), 61.0**  **49.8 (21.3), 46.0**  **0 (0.0%)**  **0 (0.0%)**  **241 (100.0%)**  **226 (93.8%)**  **220 (91.3%)**  **32 (13.3%)**  **17 (7.1%)** | **0 (0.0%)**  **22 (9.1%)**  **22 (9.1%)**  **0 (0.0%)**  **16 (6.6%)**  **9 (3.7%)**  **11 (4.6%)**  **31 (12.9%)**  **0 (0.0%)**  **0 (0.0%)**  **0 (0.0%)**  **15 (6.2%)**  **44 (18.3%)**  **60 (24.9%)**  **0 (0.0%)**  **0 (0.0%)**  **0 (0.0%)**  **0 (0.0%)**  **0 (0.0%)** |

∗ Multiple answers possible

**6MWD:** 6 min walk distance**; BMI**: body mass index; **BNP**: B-type natriuretic peptide; **CHD:** congenital heart disease; **CI:** cardiac index; **CTD:** connective tissue disease**; DLCO (% pred.):** diffusing capacity factor of the lung for carbon monoxide (predictive value**); ERA**: endothelin receptor antagonist; **FC**: functional class; **HIV:** Human Immunodeficiency Virus;  **NT-proBNP:** N-terminal pro–B-type natriuretic peptide; **PDE5i:** phosphodiesterase-5 inhibitor; **PCA**, prostacyclin analogs; **PRA**, prostacyclin receptor agonist; **PAH:** pulmonary arterial hypertension; **PAP**: pulmonary artery pressure; **PAWP**: pulmonary arterial wedge pressure**, PVOD/PCH:** pulmonary venoocclusive disease / pulmonary capillary haemangiomatosis; **RAP:** right atrial pressure**; PVR (WU):** pulmonary vascular resistance (wood units**);** **sGCs**, stimulator of soluble guanylate cyclase (Riociguat); **SvO_2:_** oxygen saturation

| Table S2: FC, 6MWD, NT-proBNP, risk status and therapy switch from baseline to first follow-up / drug class at first follow up: matched patients / patients without matching partners / all patients with comorbidities |
| --- |

***Table S2: FC, 6MWD, NT-proBNP, risk status and therapy switch / drug class from baseline to first follow-up***

| characteristics | matched patients with  comorbidities (n=216)  Mono therapy  Mean (SD), Median  or No. (%) | Missing (%) | matched patients  with comorbidities (n=216)  Combination therapy  Mean (SD), Median  or No. (%) | Missing (%) | patients without matching  partner with comorbidities  (n=1030)  Mono therapy  Mean (SD), Median  or No. (%) | Missing (%) | patients without  matching partner  with comorbidities  (n=25)  Combination thera-  py Mean (SD),  Median or No. (%) | Missing (%) | all patients with comorbidities  (n=1246)  Mono therapy  Mean (SD), Median  or No. (%) | Missing (%) | all patients with comorbidities  (n=241)  Combination therapy  Mean (SD), Median  or No. (%) | Missing (%) |
| --- | --- | --- | --- | --- | --- | --- | --- | --- | --- | --- | --- | --- |
| FC at baseline  I  II  III  IV  FC at first follow-up  I  II  III  IV  6MWD (m) at baseline  6MWD (m) at first follow-up  NT-proBNP (ng/L) at baseline  NT-proBNP (ng/L) at first follow-up | **0 (0.0%)**  **29 (13.4%)**  **156 (72.2%)**  **31 (14.4%)**  **2 (1.2%)**  **39 (22.5%)**  **115 (66.5%)**  **17 (9.8%)**  **279.9 (130.3), 283.0**  **321.1 (118.4), 330.0**  **3492 (6914), 1850**  **2084 (4251), 879** | **0 (0.0%)**  **43 (19.9%)**  **63 (29.2%)**  **70 (32.4%)**  **90 (41.7%)**  **98 (45.4%)** | **0 (0.0%)**  **29 (13.4%)**  **156 (72.2%)**  **31 (14.4%)**  **7 (3.9%)**  **58 (32.0%)**  **104 (57.5%)**  **12 (6.6%)**  **272.6 (136.7), 275.0**  **327.5 (128.7), 335.0**  **4708 (11981), 2007**  **1889 (4677), 526** | **0 (0.0%)**  **35 (16.2%)**  **57 (26.4%)**  **71 (32.9%)**  **47 (21.8%)**  **57 (26.4%)** | **3 (0.3%)**  **103 (10.0%)**  **777 (75.4%)**  **147 (14.3%)**  **11 (1.2%)**  **213 (23.0%)**  **650 (70.2%)**  **52 (5.6%)**  **276.9 (105.2), 282.0**  **310.9 (113.1), 315.0**  **2834 (4997), 1405**  **2518 (6331), 1139** | **0 (0.0%)**  **104 (10.1%)**  **225 (21.8%)**  **472 (45.8%)**  **273 (26.5%)**  **300 (29.1%)** | **0 (0.0%)**  **6 (24.0%)**  **9 (36.0%)**  **10 (40.0%)**  **1 (5.3%)**  **4 (21.1%)**  **14 (73.7%)**  **0 (0.0%)**  **244.0 (222.1), 300.0**  **358.9 (133.9), 410.0**  **4208 (6779), 2371**  **2677 (6538), 371** | **0 (0.0%)**  **6 (24.0%)**  **18 (72.0%)**  **6 (24.0%)**  **12 (48.0%)**  **4 (16.0%)** | **3 (0.2%)**  **132 (10.6%)**  **933 (74.9%)**  **178 (14.3%)**  **13 (1.2%)**  **252 (22.9%)**  **765 (69.6%)**  **69 (6.3%)**  **277.4 (109.6), 282.0**  **313.0 (114.2), 320.0**  **2928 (5314), 1491**  **2457 (6084), 1077** | **0 (0.0%)**  **147 (11.8%)**  **288 (23.1%)**  **542 (43.5%)**  **363 (29.1%)**  **398 (31.9%)** | **0 (0.0%)**  **35 (14.5%)**  **165 (68.5%)**  **41 (17.0%)**  **8 (4.0%)**  **62 (31.0%)**  **118 (59.0%)**  **12 (6.0%)**  **271.4 (140.4), 277.5**  **331.1 (129.3), 342.5**  **4673 (11675), 2038**  **1981 (4914), 505** | **0 (0.0%)**  **41 (17.0%)**  **75 (31.1%)**  **77 (32.0%)**  **59 (24.5%)**  **61 (25.3%)** |
| Risk at baseline  Low  Intermediate-low  Intermediate-high  High  Risk at first follow-up  Low  Intermediate-low  Intermediate-high  High | **10 (4.6%)**  **31 (14.4%)**  **108 (50.0%)**  **67 (31.0%)**  **18 (9.9%)**  **52 (28.7%)**  **79 (43.6%)**  **32 (17.7%)** | **0 (0.0%)**  **35 (16.2%)** | **10 (4.6%)**  **31 (14.4%)**  **108 (50.0%)**  **67 (31.0%)**  **32 (16.8%)**  **69 (36.3%)**  **63 (33.2%)**  **26 (13.7%)** | **0 (0.0%)**  **26 (12.0%)** | **17 (1.8%)**  **209 (21.6%)**  **521 (53.9%)**  **219 (22.7%)**  **68 (7.7%)**  **241 (27.2%)**  **358 (40.5%)**  **218 (24.6%)** | **64 (6.2%)**  **145 (14.1%)** | **1 (7.7%)**  **3 (23.1%)**  **2 (15.4%)**  **7 (53.8%)**  **5 (22.7%)**  **8 (36.4%)**  **7 (31.8%)**  **2 (9.1%)** | **12 (48.0%)**  **3 (12.0%)** | **27 (2.3%)**  **240 (20.3%)**  **629 (53.2%)**  **286 (24.2%)**  **86 (8.1%)**  **293 (27.5%)**  **437 (41.0%)**  **250 (23.5%)** | **64 (5.1%)**  **180 (14.4%)** | **11 (4.8%)**  **34 (14.8%)**  **110 (48.0%)**  **74 (32.3%)**  **37 (17.5%)**  **77 (36.3%)**  **70 (33.0%)**  **28 (13.2%)** | **12 (5.0%)**  **29 (12.0%)** |
| Drug class received at first follow up  ERA  PDE5i  PCA  sGC  No therapy switch (from baseline to first Follow up)  Switch from mono to combination  Therapy (from baseline to first Follow up)  Switch from combination to mono therapy (from baseline to first Follow up)  Switch to no therapy (from baseline to first follow up) | (99 (45,8%)  148 (68,5%)  6 (2,8%)  12 (5,6%)  154 (71,3%)  53 (24,5%)    –  9 (4.2%) |  | 189 (87,5%)  188 (87,0%)  49 (22,7%)  20 (9,3%)  198 (91,7%)    –  15 (6,9%)  3 (1,4%) |  | – | – | – | – | 204 (16,4%)  1006 (80,7%)  25 (2,0%)  38 (3.0%)  973 (78,1%)  231 (18.5%)    –  42 (3,4%) |  | 226 (93,8%)  213 (88,4%)  56 (23,2%)  20 (8,3%)  220 (91,3%)      –  18 (7,5%)  3 (1,2%) |  |

**6MWD:** 6 min walk distance**; FC**: functional class; **NT-proBNP:** N-terminal pro–B-type natriuretic peptide, **PDE5i:** phosphodiesterase-5 inhibitor; **PCA**, prostacyclin analogs; **sGC**, stimulator of soluble guanylate cyclase (Riociguat)
